# Insight into the Relationships Between Chemical, Protein and Functional Variables in the PBP/GOBP Family in Moths Based on Machine Learning

**DOI:** 10.3390/ijms26052302

**Published:** 2025-03-05

**Authors:** Xaviera A. López-Cortés, Gabriel Lara, Nicolás Fernández, José M. Manríquez-Troncoso, Herbert Venthur

**Affiliations:** 1 Department of Computer Sciences and Industries, Universidad Católica del Maule, Talca 3466706, Chile; 2 Centro de Innovación en Ingeniería Aplicada (CIIA), Universidad Católica del Maule, Talca 3466706, Chile; gabriel.lara@alu.ucm.cl (G.L.); nicolas.fernandez@alu.ucm.cl (N.F.); manriquez.josematias@gmail.com (J.M.M.-T.); 3 Laboratorio de Química Ecológica, Departamento de Ciencias Químicas y Recursos Naturales, Facultad de Ingenieria y Ciencias, Universidad de La Frontera, Temuco 4811230, Chile; 4 Centro de Investigación Biotecnológica Aplicada al Medio Ambiente (CIBAMA), Universidad de La Frontera, Temuco 4811230, Chile

**Keywords:** chemical ecology, lepidoptera, odorant-binding proteins, artificial intelligence, ligand binding, regression algorithm

## Abstract

During their lives, insects must cope with a plethora of chemicals, of which a few will have an impact at the behavioral level. To detect these chemicals, insects use several protein families located in their main olfactory organs, the antennae. Inside the antennae, odorant-binding proteins (OBPs), as the most studied protein family, bind volatile chemicals to transport them. Pheromone-binding proteins (PBPs) and general-odorant-binding proteins (GOPBs) are two subclasses of OBPs and have evolved in moths with a putative olfactory role. Predictions for OBP–chemical interactions have remained limited, and functional data collected over the years unused. In this study, chemical, protein and functional data were curated, and related datasets were created with descriptors. Regression algorithms were implemented and their performance evaluated. Our results indicate that XGBoostRegressor exhibits the best performance (*R*^2^ of 0.76, RMSE of 0.28 and MAE of 0.20), followed by GradientBoostingRegressor and LightGBMRegressor. To the best of our knowledge, this is the first study showing a correlation among chemical, protein and functional data, particularly in the context of the PBP/GOBP family of proteins in moths.

## 1. Introduction

Insects play an important role in ecosystems. However, as a result of globalization, invasive species have spread quickly and are now a problem in many countries [1,2]. These insect pests have an extraordinary sense of olfaction, adapting to new regions and climates using plants as hosts for feeding or oviposition [3]. In particular, moths have become serious pests throughout the world, where the cotton leaf worm *Spodoptera littoralis*, spongy moth *Lymantria dispar*, codling moth *Cydia pomonella*, oriental fruit moth *Grapholita molesta*, Indian meal moth *Plodia interpunctella*, and grapevine moth *Lobesia botrana* are few examples of highly invasive widespread polyphagous species [4].

Olfaction-driven behaviors in moths have proven to be key for the development of traps baited with odorants, either sex pheromones or attractants (i.e., semiochemicals) [5]. Traditionally, these chemicals have been identified by time-consuming methods using live insects, volatile trapping in polymers, chromatographic analysis, and, ultimately, behavioral assays in both the laboratory and field. In addition, pheromone identification has remained elusive in some species following this traditional approach [6]. More worryingly, new insects are being introduced in new countries, and pest management strategies must be implemented, involving time and expenses for local governments. Normally, insecticides are the primary and cheapest resource for insect control, and are sometimes complemented by odorant-baited traps taking advantage of the well-tuned olfactory system of moths [7]. In that sense, key odorants that elicit behavioral responses in these species must be identified [8].

In insects, the primary olfactory organs are sensilla on the antennae, inside which some protein families, called chemosensory proteins, play pivotal roles in detecting odorants (i.e., volatile organic compounds or VOCs) [9,10,11,12]. The odorants that bind to these proteins are highly specific, and in moths, it has been shown that behavioral responses can be elicited at very low concentrations (i.e., micro- or nanomolar) [13]. The first chemosensory protein was discovered in 1981 from antennae of the giant moth *Antheraea polyphemus*, and called the odorant-binding protein (OBP) [14]. More than forty years later, OBPs have become the most studied chemosensory protein and the target of choice for studying insect chemosensation, evidenced by multiple review articles [8,10,12,15,16,17,18]. Briefly, OBPs transport odorants from olfactory pores located in hair-like structures called sensilla, which are distributed across antennae. Afterwards, OBPs deliver odorants to ORs for olfactory transduction, and, ultimately, insect behavioral responses are unleashed. In moths, an evolutionarily conserved clade of OBPs are significantly expressed in antennae rather than other tissues, showing high binding affinities (i.e., K_i_) to odorants with semiochemical functions [19,20,21].

Nowadays, genomic and transcriptomic approaches allow the identification of dozens to hundreds of insect moth OBPs, with 15–45 OBPs usually identified in each lepidopteran species [16,22]. OBPs, from olfaction-derived data, are considered the first filter of odorants in the antennae of insects, and are extensively studied [22]. To date, approximately 28,700 amino acid sequences for OBPs have been deposited in UniProt database. This is 2.3 times the number of sequences for ORs, the other olfactory proteins that recognize odorants in insects [23]. Although recent evidence suggests that OBPs appear to have both chemosensory and non-chemosensory functions, OBPs still are crucial for insect olfaction [12].

Furthermore, OBPs have become the target of choice for odorant discovery due to their inherent binding affinities [10,16]. The use of OBPs for in vitro functional evidence can overcome factors related to live insects, such as life cycle, size, abundance, and colony ñrearing. Furthermore, ample sets of chemicals can be used, accelerating the identification of odorants with behavioral effects. For instance, the fluorescence binding assay (an in vitro assay) has become frequent method to test the binding affinity of VOCs to insect OBPs, resulting in inhibition constant (K_i_) values measured in the nano- or micro-molar (nM or μM, respectively) range [16,24,25]. Currently, 215 functional studies combining OBPs and VOCs have been reported, and subsequently, 622 VOCs have been counted with quantifiable data through initiatives such as iOBPdb [26], a centralized database that reunites OBP and VOC information along with their binding affinities. In moths, a particular evolutionary clade occurs with OBPs named general-odorant-binding proteins (GOBPs) and pheromone-binding proteins (PBPs), which are highly conserved among lepidopterans [27,28]. Although increasing evidence suggests that OBPs might play other chemosensory and non-chemosensory roles, research has shown that PBPs and GOBPs are still crucial in transporting sex pheromone components or both plant volatiles and sex pheromones [12,28,29,30,31]. Currently, it is known that moths have three types of PBPs, whereas butterflies have two, probably because of the nocturnal habits in moths and, therefore, their odorant-guided behaviors [28]. Likewise, it is common to identify two GOBPs in moths [16,32].

**Figure 1:**
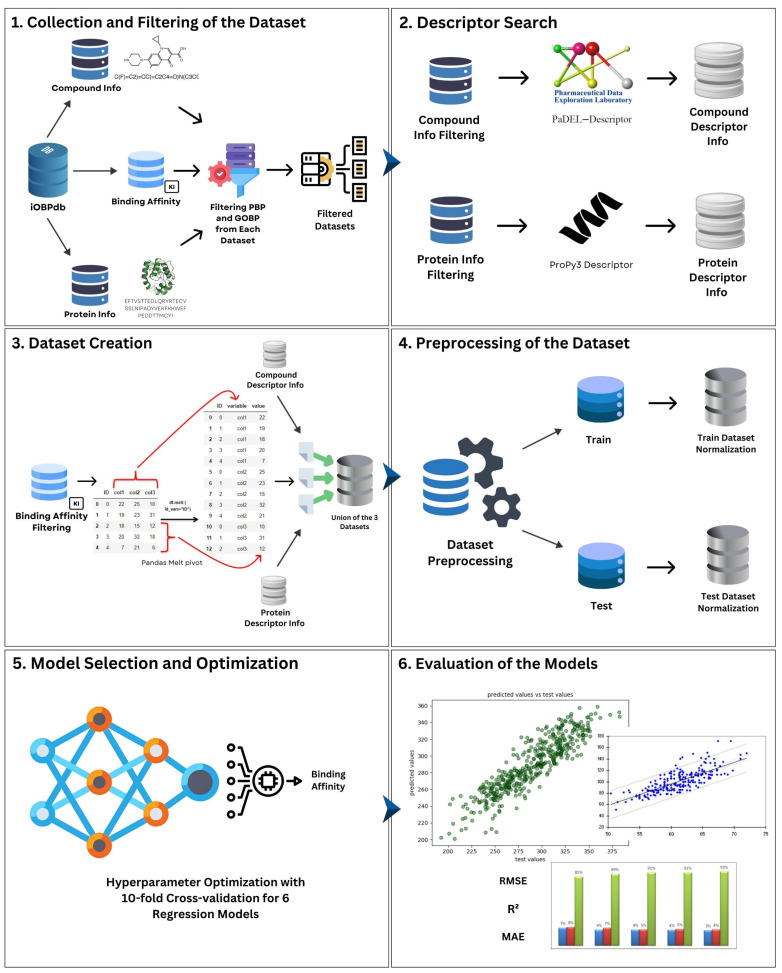
Scheme of proposed methodology. (**1**) Collection and filtering of PBP and GOBP related dataset. (**2**) Descriptor search based on amino acid sequences and SMILES of compounds. (**3**) Dataset creation of protein and chemical descriptors along with their respective binding affinity. (**4**) Cluster of data divided into 80% training and 20% testing, and normalization of StandardScaler characteristics followed by transformation of binding affinities for each cluster. (**5**) Implementation of 6 ML regression models: For training, optimization for hyperparameters with ten-step cross-validation for each ML was used. (**6**) For testing, training with best parameters for 6 ML regression models was used in order to evaluate the results on plots as shown in the circles in the figure and compare performance according to Root-Mean-Square Error (RMSE), Coefficient of Determination (*R*^2^) and Mean Absolute Error (MAE).

Considering the above, current initiatives have focused on comprehensively studying the main olfactory organ of insects (i.e., antennae), and related proteins that bind VOCs, providing an opportunity to identify novel behaviorally active chemicals and, consequently, use them in pest management. Hitherto, molecular and bioinformatics approaches have addressed the above with some success, where OBPs have played a role as targets [16]. However, new cheaper, reproducible and scalable methodologies are needed to identify odorants with the potential to be implemented in pest management. In this sense, advances in computer science have resulted in software capable of learning, helping in visual perception, translation between languages, speech recognition and decision-making tasks, i.e., artificial intelligence (AI) [33]. Applications of AI to biological problems are becoming powerful methods of solving biological problems at different scales [34,35,36,37,38]. One type of AI enables a computer to learn on its own, and this is called machine learning (ML). ML can identify patterns from databases and make predictions [39,40,41,42]. Nowadays, most of the research that integrates ML and pest management seems to be focused on identification and monitoring rather than control [43]. Thus, ML has been applied to monitoring insects’ flight based on their dependence on abiotic factors, such as temperature, wind, humidity, etc. Interestingly, a neural network method with four layers (a type of ML model) was developed to track the flight of the grapevine moth L. botrana, which is highly influenced by temperature [44]. Likewise, a 79% accuracy in predicting thrip and squamous and black weevil incidence using a supervised ML algorithm in the form of logistic regression and vector machine has been reported [45]. One step further was reported for the noctuid moth *S. littoralis*, whereby the authors found the chemical space of volatiles that could elicit behavioral activity (attraction or repellency) based on the activation of odorant receptors (ORs) (i.e., SlitOR25) from a panel of 3 million compounds and using a QSAR model (as a supervised ML model) [46]. Despite the suitability of using ML models for the identification of behaviorally active odorants, current reports are from a chemical perspective. Also, sequence-based predictions have been investigated, and to the best of our knowledge, function-based predictions through ML have not been considered for the previously mentioned purposes. The closest approach to this has been through AlphaFold2 (a deep learning-based tool), predicting the 3D structure of olfactory proteins and identifying odorants with putative biological activity [47,48,49]. However, these studies have focused on binding characterization from a structural perspective only, and final applications (e.g., traps baited with new odorants) are still lacking. Therefore, the objective of this study was to evaluate and select suitable ML models that can integrate not only chemical and sequence descriptors (i.e., odorants and OBPs, respectively), but also functional data in the form of the inhibition constant *K_is_*, which represents the binding affinity between proteins and ligands, but focusing on PBPs and GOBPs from moths. Here, a proposed methodology consisted in six main steps with collection and filtering of data, descriptor search, dataset creation, preprocessing of datasets, model selection and optimization and evaluation (Figure 1). 

## 2. Results

**Figure 2:**
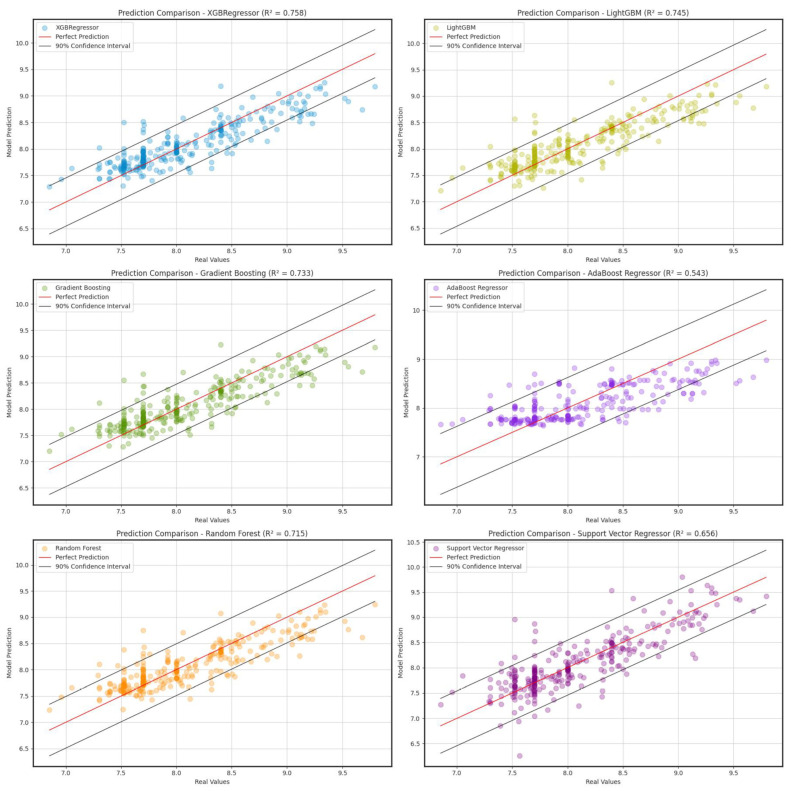
A Comparison of the predictions with the 20% test data using different regression models. The actual values used are from the iOBPdb database [26].

The performance of the optimized models was evaluated using a cluster test (20% of the data), with the best parameters found through the Root-Mean-Square Error (RMSE), Coefficient of Determination (*R*^2^) and Mean Absolute Error (MAE) [50]. The results from the metrics of each model are presented in Table 1 and Figure 2.

In terms of accuracy, the XGBoostRegressor model showed the best performance, reaching an *R*^2^ of 0.758, with an RMSE of 0.276 and an MAE of 0.202. This model presented the best predictions in comparison with the real values, which positions it as a suitable curated dataset. It was closely followed by GradientBoostingRegressor and LightGBMRegressor, which showed an R^2^ of 0.733 and 0.745, respectively. Both models presented similar RMSE and MAE values when compared with XGBRegressor, although with a slight decrease in accuracy.

The RandomForestRegressor also showed a satisfactory yield, with an R^2^ of 0.715 and an RMSE of 0.300, which suggests a robust capacity for predictions, although inferior to other boosting methods such as XGBoostRegressor, GradientBoostingRegressor or LightGBMRegressor. On the other hand, Support Vector Regressor (SVR) obtained an *R*^2^ of 0.656, indicating a lower adjustment capacity in comparison with previous models.

Finally, AdaBoost Regressor presented the lowest performance of all the evaluated models in this study, with an *R*^2^ of 0.543 and higher values of RMSE and MAE, suggesting a limited capacity to capture the relationships among variables (chemical, protein and functional).

The scatter plots (Figure 2) compare the actual values with the values predicted by each model. The 90% confidence intervals are included to assess the accuracy of the predictions. XGBoost and LightGBM show strong alignment with the perfect prediction line (red line), confirming their high predictive power. Gradient Boosting and Random Forest also present good fits, albeit with a slight dispersion. AdaBoost and SVR exhibit higher errors and higher variability in their predictions, which is reflected in their lower R^2^ values.

Table 1 and Figure 2 illustrate the superiority of XGBoostRegressor in predicting affinities with fewer errors and closer to a perfect prediction line, followed by LightGBMRegressor and GradientBoostingRegressor. On the other hand, AdaBoostRegressor is not recommended due to its high error rate and low fit. These findings suggest that decision tree-based methods with boosting are the most effective for this problem.

**Figure 3:**
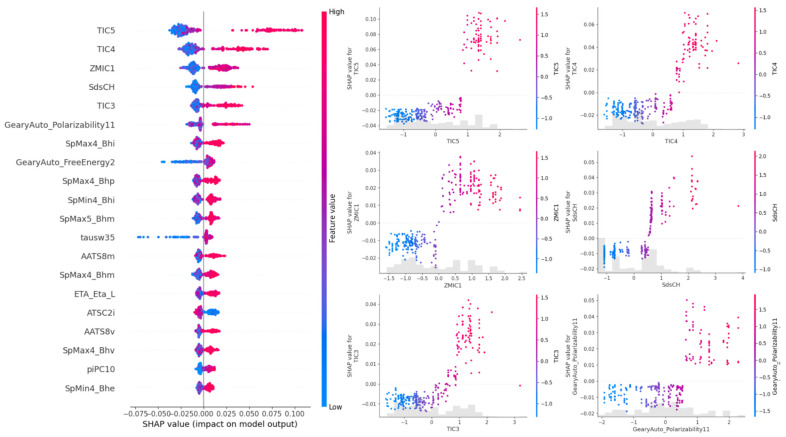
A SHAP (Shapley Additive exPlanations) summary plot and individual feature impact plots for the XGBoostRegressor model. The left panel shows the global importance of the features ranked by their impact on the model’s prediction of binding affinity (*K_i_*) between OBPs (PBP/GOBP family) and VOCs. Each dot represents a SHAP value for a specific interaction, with colors indicating the feature value (red: high; blue: low). The right panel presents detailed SHAP dependency plots for the five most impactful features (TIC5, TIC4, ZMIC1, SdsCH and TIC3), highlighting how variations in these descriptors influence the predicted binding affinity. The gray histograms in the dependency plots represent the distribution of feature values in the dataset.

In order to evaluate the feature contribution, the model with the best performance for *K_i_* prediction was used. Specifically, the XGBoost model was used to conduct Shapley value analysis. Figure 3 shows the Shapley values of the 20 features (descriptors obtained with PaDEL or Propy3) with the highest average contribution for *K_i_* prediction. Specifically, the features TIC5, TIC4, ZMIC1, SdsCH and TIC3 correspond to molecular descriptors calculated using the PaDEL-Descriptor tool. Specifically, TIC5, TIC4 and TIC3 refer to the Total Information Content Index—Neighborhood Symmetry of the *n*-th order. These descriptors quantify the total structural information based on the symmetry of atoms within the molecule at different neighborhood levels (n-th order). Since the SHAP analysis shows that TIC5, TIC4 and TIC3 are the most influential features, this suggests that the structural symmetry of the molecule at different neighborhood levels is key for interaction with OBP/PBP proteins. Similarly, ZMIC1 (Z-modified Information Content Index of the first order) is a modified variant of the Information Content Index (TIC), adjusted with the Z constant, and captures the first-order neighborhood symmetry of the molecule. Finally, the SdsCH descriptor is an electrotopological descriptor that belongs to the atom-type descriptors of the electrotopological state (E-State). These descriptors are used in cheminformatics to quantify the electronic and topological properties of specific atoms within a molecule. In this case, SdsCH specifically refers to the sum of the electrotopological state values for =CH-type atoms. The fact that these features appear in the top five SHAP values could indicate a strong correlation with the prediction of the K_i_ value.

## 3. Discussion

Olfaction plays a crucial role in the life cycle of an insect. Over time, research has provided insights into how odorants are recognized by a well-tuned olfactory system, especially in lepidopterans, where it all started. In 1959, the first sex pheromone was identified from the silk moth *Bombyx mori* [50,51]. To date, more than 6500 compounds have been identified and deposited in the Pherobase database [52] that mediate interactions among insects, whether sex pheromones (intraspecific) or allelochemicals (interspecific), both classed as semiochemicals [53]. Some of these chemical compounds, usually volatiles, are currently used in field traps for monitoring and control. However, their discovery is highly demanding in terms of time and laboratory expenses and dependent on insect availability. Therefore, alternative strategies that can overcome these difficulties and, at the same time, use data from insect olfaction are necessary.

Hitherto, research has focused on insect olfaction following two pathways. The first is studying chemical information from odorants with or without semiochemical function. For example, a supervised ML model through QSAR was used to screen a panel of 3 million compounds that could elicit behavioral activity (attraction or repellency) in the moth *S. littoralis* based on the activation of OR25 [54]. Similarly, a novel set of antagonistic volatile compounds were reported for Orco (a conserved insect OR co-receptor) from the fruit fly *Drosophila melanogaster* using ML models, such as a Naïve Bayesian classifier and Extended Connectivity Fingerprints. Their results suggested 2-tert-butil-6-metilfenol was the best at inhibiting behavioral responses in larvae of *D. melanogaster* [55]. In this sense, chemical, structural and functional descriptors appear to be key in decoding the odorant–protein relationship. Thus, SMILES notations have been used for finding odorant–smell relationships through deep learning approaches, such as deep neural networks (DNN) and convolutional neural networks (CNN) [56]. Likewise, the use of chemical descriptors has provided predictions of odor perception based on chemical structure [57]. ML has also been applied for OBP sequence classification through the Regularized Least-Squares Classifier (RLSC) [58]. Hence, chemicals and proteins have been analyzed independently, and therefore, the odorant–OBP relationship has not been decoded by ML algorithms.

A third pathway could be the implementation of functional properties derived from the binding of a given odorant to an OBP (i.e., *K_i_*) and evaluated through ML algorithms. To the best of our knowledge, this approach has remained unexplored. In this study, three datasets were unified and implemented under supervised ML. Thus, XGBoostRegressor resulted in an *R*^2^ of 0.758, and a prediction of the binding between OBPs and ligands by combining, for the first time, both chemical and protein descriptors along with functional data in the form of K_i_. These findings suggest that the prediction of binding affinities in the context of OBPs is feasible. The functional properties from OBPs in moths, particularly PBPs and GOBPs, have been obtained through fluorescence-based assays. Here, chemical compounds (such as odorants) displace *N*-phenyl-1-napthylamine (1-NPN, also called a fluorescent probe) from the unique binding site present in OBPs, acting as competitors. Although a comparative study reported that K_i_ could change depending on the fluorescent probe, this competitive fluorescent assay is still the most widely used technique for insect OBPs [16,59]. Consequently, initiatives such as iOBPdb, as a database that reunites VOCs (or odorants as ligands), OBPs and the resulting binding affinities (i.e., K_is_), have emerged [26]. Thanks to this database, it is possible to download current deposited data related to OBPs, VOCs and Kis separately. Furthermore, researchers can contribute their own data, helping to constantly update iOBPdb [60]. Notably, outside the insect olfaction field, other studies have used unified functional and chemical data for ML algorithms. An example is the identification of two lactones as potential inhibitors of acetylcholinesterase (AChE), an important target of research in Alzheimer’s disease, found based on 7032 molecules with IC_50_ and another 8593 secondary metabolites through classification models [61]. Similarly, datasets of cytochrome P450 inhibitors and IC_50_ were implemented for Random Forest and SVM, resulting in over 80% accuracy [62].

With respect to the models implemented, it is possible to say that the best performance in predicting *K_i_* corresponded to the XGBoostRegressor model, which outperformed LightGBMRegressor, GradientBoostingRegressor, AdaBoostRegressor, RandomForestRegressor and SupportVectorRegressor. The Shapley value analysis (3) revealed that most of the features with high predictive contributions were derived from the ligand, specifically the PaDEL-Descriptors. TIC descriptors measure the topological structural complexity of a molecule, while ZMIC evaluates its connectivity based on Zagreb indices. Among the top five contributors to the prediction of *K_i_*, it is worth noting that TIC5, TIC4, ZMIC1 and TIC3 represent ligand descriptors. These indices capture key aspects of molecular structural diversity, including electrical, geometric, symmetric and topological properties. This finding underscores the critical role of ligand structural features in influencing binding affinity predictions. Moreover, the SdsCH descriptor, which pertains to protein-specific characteristics, particularly hydrophobicity, further highlights the importance of protein–ligand interactions in the predictive model. The integration of both ligand-centric and protein-specific features suggests a multifaceted approach to modeling, where the roles of the structural and physicochemical properties of both entities are essential for accurate predictions. These observations provide valuable insights into the mechanisms driving protein–ligand interactions, particularly the binding of PBPs and GOBPs to VOCs, and pave the way for refining future computational models.

Although the implemented models yielded promising results, due to the predictive power obtained through *R*^2^, there are several avenues for enhancing the proposed approach. First, increasing both the volume and the diversity of the data could significantly improve the generalizability of the model [63]. Likewise, expanding the dataset by incorporating additional OBP and VOC protein data from various databases would extend the analysis to encompass a broader range of species and chemical compounds. Moreover, performance evaluation could be beneficial for the inclusion of alternative metrics, such as the Mean Absolute Percentage Error (MAPE), Concordance Index (CI) or domain-specific metrics like Receive Operating Characteristic (ROC) and Regression Error Characteristic Curves (REC), providing deeper insights into critical protein–ligand interactions.

Another potential enhancement involves adopting periodic retraining techniques to account for new data or leveraging transfer learning models to capitalize on prior knowledge from related problems. Furthermore, exploring advanced neural network architectures, such as DeepDTA or Affinity2Vec [64,65], could better capture the intricate complexities of protein–chemical interactions. Finally, employing advanced feature engineering strategies, such as dimensionality reduction tailored to domain-specific relationships or custom feature creation, can optimize data representation and improve predictive accuracy [66,67].

These advances will not only enhance the robustness of the model, but also broaden its applicability in future studies on ecological chemistry and integrated pest management.

## 4. Materials and Methods

### 4.1. Data and Preproccessing

#### 4.1.1. Data

The dataset was extracted from the iOBPdb database [26], a free bioinformatics re- source containing information on odorant-binding proteins (OBPs), volatile organic compounds (VOCs) and the interaction affinity between them. In particular, three specific subsets of data were used:

- Odorant-binding proteins

This database contains information on 436 OBPs such as their names, species, cystine counts, protein types, and amino acid sequences with and without signal peptides, among other characteristics.

- Volatile organic compounds

This database contains information on 621 VOCs such as their names and properties like molecular formulas, SMILES and functional groups to which they belong.

- Binding affinity (K_i_)

This database contains a 621 × 436 matrix that records the binding affinity (*K_i_*) between VOCs and OBPs.

#### 4.1.2. Preprocessing

**Figure 4:**
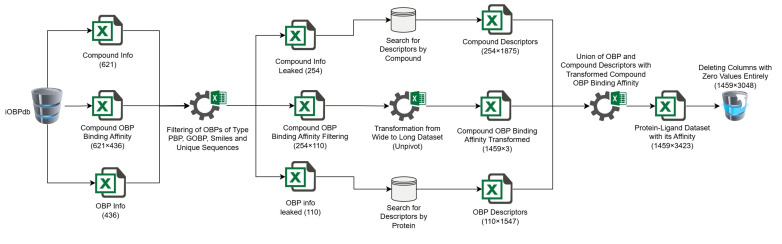
Flow chart scheme of proposed methodology (available on the Supplementary Material).

The process of filtering and transforming the iOBPdb data began by selecting OBPs only from species in the order Lepidoptera that fit the PBP and GOBP subcategories, applying a taxonomic filter to exclude non-Lepidopteran species using the Python Pandas library [68,69]. Next, volatile organic compounds (VOCs) related to these species were identified, ensuring that only VOCs directly linked to Lepidoptera remained. The binding affinity values for each protein–ligand pair were then extracted from iOBPdb (https://www.iobpdb.com (accessed on 17 April 2024)) [26,60], providing key interaction data between odorant-binding proteins (PBPs and GOBPs) and VOCs. To eliminate redundancy, duplicate SMILES structures and repeated amino acid sequences were identified and removed, ensuring the uniqueness of each protein–ligand pair. This resulted in three new sets of 110 OBPs, 254 VOCs and a 254 × 110 affinity matrix reflecting the binding affinity (K_i_) of each protein–ligand combination (Figure 4).

#### 4.1.3. Extraction of Descriptors

For the 254 VOCs, using the PaDEL-Descriptor library [70] (through PaDEL-Py, its Python 3.9 implementation [71]), 1875 descriptors were generated (431 3D features and 1444 2D and 1D features) per compound, including physicochemical, topological, geometrical and other characteristics. For the 110 OBPs generated through Python’s PyPro3 [72] library, 1547 descriptors were calculated based on the amino acid sequence without signal peptides, such as amino acid composition, physicochemical properties, hydrophobicity and other relevant characteristics.

#### 4.1.4. Dataset Creation

The affinity matrix was transformed into a long format, where each row represents a single VOC-OBP interaction with its binding affinity value (*K_i_*), yielding a dataset of 1459 compound–protein interactions. Two merges were performed to enrich the dataset with descriptors of VOCs and OBPs with the Merge function of the Python Pandas library [68,71]. Features with zero were then removed in their entirety, generating a final set of 1459 interactions and 3048 descriptor features.

#### 4.1.5. Machine Learning Models

- Training and testing data.

To assess the generalizability of the models, the dataset was split into 80% (1167 × 3048) for training and 20% (292 × 3048) for testing with a seed of 41 to ensure reproducibility. The training subset was used to fit and optimize the models, while the test set allowed performance to be assessed on unseen data, ensuring a robust measure of their accuracy.

- Dataset preprocessing

Considering variation in data scales and ranges, normalization was applied to improve the performance and stability of the models. In particular, the StandardScaler method of the Scikit-Learn library [73] was used, which transforms each characteristic according to the mean and standard deviation of its values, allowing a normalized distribution to be obtained with a mean of 0 and a standard deviation of 1:

(1) $$X_{scaled}=\frac{X-\mu }{\sigma }$$

where the definitions are as follows:

- *X* is the original value of the feature;
- *μ* is the mean of the feature in the dataset;
- *σ* is the standard deviation of the feature.

In addition, the binding affinity (*K_i_*) values were transformed into a logarithmic scale (*pK_i_*), similar to the SimBoost, DeepDTA and Affinity2Vec [54,65,66,67,68,69,70,71,72,73] methods, by applying the following equation:

(2) $$pKi=-log1\left(\frac{K_{i}}{1\times 10^{9}}\right)$$

- *K_i_* represents the value of the inhibition constant in units of molarity (M).
- The factor $1\times 10^{9}\;$(or 10^9^ nM) is used to convert *Ki* to nanomolarity (nM) so that the resulting logarithmic values are on a comparable scale.

This transformation converts the value of *K_i_* into a scale that is easier to interpret. These values in the dataset range from 6.30 to 9.80, where high values of *pK_i_* indicate a strong binding affinity (i.e., lower *K_i_*) and low values indicate a weak affinity. To obtain the affinities on their original scale, just apply the inverse function (from *pK_i_* to *K_i_*) to convert the *pK_i_* values predicted by the model back to molarity units. The inverse formula to recover *K_i_* from *pK_i_* is

(3) $$K_{i}=10^{9-pK_{i}}$$

### 4.2. Models’s Implementation

The regression algorithms implemented in this study corresponded to supervised learning, where the following regression models were applied: Gradient Boosting (GB), AdaBoost (AB), Random Forest (RF) and Support Vector Regressor (SVR) [73]. XGBRegressor from the XGBoost library [74] was also applied, as was LGBMRegressor from the LightGBM library [75]. A brief theoretical description of each is given below:

- XGBoost Regressor

This algorithm represents an advanced decision tree-based boosting method designed to continuously improve its predictions. This model adjusts multiple trees in sequence, with each tree attempting to correct the errors of its predecessors. XGBRegressor’s ability to perform fine-tuning, through gradient optimization, allows for excellent accuracy in complex regression problems. Its flexibility and robustness to overfitting make it a preferred choice for high-dimensional regression scenarios and heterogeneous data.

- LightGBM Regressor

This is a boosting algorithm that is based on decision trees and distinguished by its unique focus on growing leaves instead of levels. This approach allows for greater accuracy in less time, optimizing memory usage and reducing training times. Its ability to handle large data volumes and high dimensionality makes it especially valuable in regression problems where a balance between speed and accuracy is sought. In addition, its dynamic fitting and sparse data handling capabilities position it as an effective tool in advanced applications.

- Gradient Boosting Regressor

This is a sequential boosting algorithm that optimizes performance by combining multiple simple models. Each subsequent model corrects the errors of the previous one through gradient descent, allowing for a steady improvement in prediction accuracy. This model is especially effective in regression problems that require a high level of accuracy and is able to capture complex relationships in the data without extensive pre-processing.

- AdaBoost Regressor

This is an iterative boosting algorithm that consecutively fits simple models, paying more attention to mispredicted observations at each iteration. This method allows for continuous model fitting, resulting in improved accuracy without the need for complex configurations. AdaBoost is a reliable choice in regression applications where an adaptive and fast-fitting model is needed, providing effective solutions to problems of moderate complexity.

- Random Forest Regressor

This is an ensemble algorithm that builds multiple decision trees and averages their results to obtain a stable and accurate prediction. This model is highly effective in reducing overfitting and is especially valuable for handling high-dimensional data and noise. Its ability to maintain a balance between accuracy and efficiency makes it a reliable and versatile tool in a wide range of regression applications.

- Support Vector Regressor

This is a support vector machine-based algorithm designed to find the optimal hyperplane that minimizes prediction error. This approach allows SVR to efficiently handle non-linear relationships and produce accurate predictions even in datasets with high variability. Thanks to its ability to control the sensitivity of the model to extreme data, SVR is a powerful tool in regression scenarios where accuracy is required under conditions of complexity and non-linearity.

#### Hyperparameter Optimization and Cross-Validation

To maximize the accuracy of the models and avoid overfitting, hyperparameter optimization was performed with 100 evals for each model along with thorough cross-validation at 80% of the data. Hyperparameter optimization was implemented using Hyperopt [76], a Bayesian optimization method that searches for the best set of parameters for each model by iteratively evaluating different combinations of hyperparameters. The search spaces for each parameter were defined in terms of appropriate ranges and values for each specific model, maximizing the accuracy and stability of the predictions.

To evaluate the performance of each set of hyperparameters, Scikit-Learn’s cross-validate feature was used with cross-validation of 10 partitions (cv = 10). This technique divides the training data into 5 subsets, where each partition is used once as a test set, while the remaining partitions are used for model training. The results obtained from each partition were averaged to obtain a global performance metric, using the RMSE, *R*^2^ and MAE [77] as the main metrics. This combination of optimization and cross-validation provides a robust model and prevents the fit from being influenced by a single test dataset, which improves the generalizability and accuracy of the final model. The optimal hyperparameters that were used in this experiment to develop the prediction models are shown in Table 2.

### 4.3. Models’ Performance Evaluation

#### 4.3.1. Root-Mean-Square Error (RMSE)

The Root-Mean-Square Error (RMSE) is a robust metric that measures the accuracy of prediction models by assessing the average deviation of predictions from actual values. This value, expressed in the same units as the target variable, facilitates direct and comparative interpretation of the error. A low RMSE [77] indicates that the model fits the data well, thus representing an accurate and reliable prediction. This metric is notable for its sensitivity to large errors, helping to identify areas for improvement in model accuracy.

(4) $$RMSE=\sqrt{\frac{1}{n}\sum_{i=1}^{n}{\left(y_{i}-{\hat{y}}_{i}\right)}^{2}}$$

- *n*: total number of observations.
- $y_{i}$: real value of the observation *i*.
- ${\hat{y}}_{i}$: predicted value for the observation *i*.

#### 4.3.2. Coefficient of Determination

The Coefficient of Determination (*R*^2^) is a metric that assesses the proportion of variability in the target variable that the model is able to explain. This indicator, expressed as a value between 0 and 1, provides a clear understanding of the effectiveness of the model. A high *R*^2^ [77] suggests a satisfactory fit, demonstrating that the model adequately captures the trends in the data.

(5) $$R^{2}=1-\frac{\sum_{i=1}^{n}{\left(y_{i}-{\hat{y}}_{i}\right)}^{2}}{\sum_{i=1}^{n}{\left(y_{i}-\bar{y}\right)}^{2}}$$

- $y_{i}$: real value of the observation *i*.
- ${\hat{y}}_{i}$: predicted value for the observation *i*.
- $\bar{y}$: mean of all real values $y_{i}$.
- *n*: total number of observations (256).

#### 4.3.3. Mean Absolute Error

The Mean Absolute Error (MAE) measures the accuracy of the model by calculating the average of the absolute differences between predictions and actual values. This metric provides a clear and direct view of model performance as it reflects the average error in absolute terms and is less sensitive to outliers. A low MAE [77] reflects the model’s ability to consistently make accurate predictions, inspiring confidence in the robustness and applicability of the model in real-world scenarios.

(6) $$MAE=\frac{1}{n}\sum_{i=1}^{n}\left|y_{i}-{\hat{y}}_{i}\right|$$

- *n*: total number of observations.
- $y_{i}$: real value of the observation *i*.
- ${\hat{y}}_{i}$: predicted value for the observation *i*.

#### 4.3.4. Confidence Interval

The Confidence Interval (CI) assesses the accuracy of the model predictions and provides a range of uncertainty in the estimates. The Confidence Interval [78,79] was calculated based on the standard deviation of the residuals. This interval allows us to estimate the range within which most predictions are expected to lie, given a specific confidence level.

This approach assumes that the residuals are approximately normally distributed and therefore allows for the construction of a symmetric confidence interval around each prediction. By plotting these intervals alongside the predictions, it is possible to visualize the expected variability in the model and to assess whether its predictions are concentrated close to the true values or whether there is high dispersion. Figure 2 compares the predictions of each model against the actual values, with a confidence interval of 90%.

### 4.4. SHAP Values

SHAP (Shapley Additive exPlanation) analysis was used to interpret the importance and impact of key features, specifically aiming to identify which chemical or protein descriptors have the greatest impact on the binding affinity predicted by the model. In this study, SHAP values were calculated for the XGBoostRegressor model, considering its performance and compatibility with the library.

## 5. Conclusions

The characterization of binding proteins such as PBPs and GOBPs, and their interaction with VOCs in lepidopterans, show their relevance in odorant detection as well as their potential application within an integrated pest management strategy. In this study, the performed analysis integrated both chemical and protein descriptors along with functional data *K_i_*, evidencing that approaches based on ML are effective tools for decoding complex protein–ligand relationships.

Notably, this study represents the first effort to focus exclusively on predicting *K_i_* binding affinity for odor-binding proteins specifically associated with pheromones and general odors in Lepidoptera. This novel approach highlights an underexplored area within protein–drug ligand research, providing a foundation for future investigations into the unique molecular interactions of these specialized proteins.

Notably, these findings highlight the value of computational methodologies to overcome some limitations in traditional experimental approaches, such as high dependency on live insects, related economical costs and challenges associated with the throughput identification of bioactive chemicals (e.g., semiochemicals). The capacity of prediction that has been demonstrated could allow the identification of novel bioactive compounds, which could be used as attractants or repellents for pest control. Finally, this study reinforces the utility of predictive models to integrate both molecular and functional data, helping to understand the depth with which insects can detect chemicals from the environment. It is believed that this approach will form the basis for future research around chemical ecology and applied biotechnology in the context of insect pest control.

## Figures and Tables

**Table 1:** Evaluation of predictions with 20% test.

Models	RMSE	*R* ^2^	MAE
**XGBoostRegressor**	**0** **.** **276**	**0** **.** **758**	**0** **.** **202**
**LightGBMRegressor**	0.284	0.745	0.208
**GradientBoostingRegressor**	0.290	0.733	0.216
**AdaBoostRegressor**	0.380	0.543	0.292
**RandomForestRegressor**	0.300	0.715	0.222
**SupportVectorRegressor**	0.329	0.656	0.236

**Table 2:** Hyperparameters explored and optimal values for models implemented.

Model	Parameters	Hyperparameter Search	Optimal Value
**XGBoostRegressor**	n_estimators	[700, 1200]	800
	learning_rate	[0.009, 0.03]	0.0188610
	max_depth	[10, 15]	10
	min_child_weight	[5, 10]	9
	gamma	[0.00, 0.005]	0.002323
	colsample_bytree	[0.3, 0.6]	0.392232
	subsample	[0.6, 0.9]	0.564263
	reg_alpha	[0.5, 1.0]	0.683823
	reg_lambda	[1.5, 2.0]	1.711287
**LightGBMRegressor**	n_estimators	[700, 1200]	900
	learning_rate	[0.009, 0.03]	0.0222877
	max_depth	[10, 20]	13
	num_leaves	[20, 150]	148
	min_child_weight	[5, 10]	7
	subsample	[0.5, 1.0]	0.661123
	colsample_bytree	[0.3, 0.8]	0.305414
	reg_alpha	[0, 2]	0.316713
	reg_lambda	[0, 3]	1.579887
**GradientBoostingRegressor**	n_estimators	[100, 500]	400
	learning_rate	[0.009, 0.03]	0.0265516
	max_depth	[5, 15]	5
	Subsample	[0.5, 1.0]	0.687177
	min_samples_split	[2, 10]	10
	min_samples_leaf	[1, 10]	1
	max_features	[0.1, 0.5]	0.260379
**AdaBoostRegressor**	n_estimators	[100, 500]	100
	learning_rate	[0.009, 0.03]	0.00996526
**RandomForestRegressor**	n_estimators	[700, 1200]	1000
	max_depth	[3, 20]	16
	min_samples_split	[2, 20]	5
	min_samples_leaf	[1, 20]	1
**SupportVectorRegressor**	C	[1000, 5000]	1000
	epsilon	[0.009, 0.03]	0.029727
	degree	[1, 15]	13

## Data Availability

Available on request from the corresponding author.

## Supplementary Materials

This supplementary material can be accessed through the link https://github.com/Glarah453/ML_obps_vocs (accessed on 18 January 2025). All files are accompanied by detailed documentation and practical examples to facilitate the reproducibility of the results. The source code, processed datasets and scripts used to reproduce the experiments presented in this study are available in the public GitHub repository “ML_obps_vocs”.
